# 3D Liver Tumor Segmentation in CT Images Using Improved Fuzzy* C*-Means and Graph Cuts

**DOI:** 10.1155/2017/5207685

**Published:** 2017-09-26

**Authors:** Weiwei Wu, Shuicai Wu, Zhuhuang Zhou, Rui Zhang, Yanhua Zhang

**Affiliations:** ^1^Faculty of Information Technology, Beijing University of Technology, Beijing 100124, China; ^2^College of Life Science and Bioengineering, Beijing University of Technology, Beijing 100124, China

## Abstract

Three-dimensional (3D) liver tumor segmentation from Computed Tomography (CT) images is a prerequisite for computer-aided diagnosis, treatment planning, and monitoring of liver cancer. Despite many years of research, 3D liver tumor segmentation remains a challenging task. In this paper, an efficient semiautomatic method was proposed for liver tumor segmentation in CT volumes based on improved fuzzy* C*-means (FCM) and graph cuts. With a single seed point, the tumor volume of interest (VOI) was extracted using confidence connected region growing algorithm to reduce computational cost. Then, initial foreground/background regions were labeled automatically, and a kernelized FCM with spatial information was incorporated in graph cuts segmentation to increase segmentation accuracy. The proposed method was evaluated on the public clinical dataset (3Dircadb), which included 15 CT volumes consisting of various sizes of liver tumors. We achieved an average volumetric overlap error (VOE) of 29.04% and Dice similarity coefficient (DICE) of 0.83, with an average processing time of 45 s per tumor. The experimental results showed that the proposed method was accurate for 3D liver tumor segmentation with a reduction of processing time.

## 1. Introduction

Liver cancer is one of the most common types of cancerous diseases worldwide, with increasingly high morbidity [1]. Early diagnosis and treatment are crucial to improve the survival rate, and the medical imaging techniques provide great help. Among many different imaging modalities, Computed Tomography (CT) is widely used for diagnosis of hepatic disease as it can provide relatively high resolution images with accurate anatomical information [2]. Three-dimensional (3D) segmentation of liver and tumors from CT images is an important prerequisite for early diagnosis, treatment planning, and monitoring of liver cancer [3]. However, manual segmentation of liver tumors slice by slice is still routinely used by radiologists in clinical practice, which is laborious and time-consuming due to the large amount of data, and also prone to interobserver variability [4]. The need for accurate and efficient tumor delineation leads to the development of semiautomatic or automatic tumor segmentation methods.

3D liver tumor segmentation remains a challenging task due to the high variability of tumor shape, size, intensity, and the low contrast between tumor and surrounding liver tissues [5]. Many different approaches have been proposed to improve the tumor segmentation performance. Recent publications mainly included semiautomatic and automatic tumor segmentation methods based on region growing or thresholding [6–8], clustering [9–11], level set [12, 13], graph cuts [14, 15], and machine learning [16–19].

Region growing, thresholding, or clustering methods have been commonly used in medical image segmentation since they are fast and easy to be implemented and have a relatively low computational cost. However, the main drawback of these methods is that only intensity information is used. As a result, such methods are prone to boundary leakage on blurred tumor boundaries. Thus, prior knowledge or other algorithms were integrated to reduce undersegmentation or oversegmentation [7, 8, 11]. Anter et al. [7] proposed an automatic tumor segmentation method using adaptive region growing. The initial seed points for region growing were detected by applying marker-controlled watershed algorithm. Zhou et al. [8] presented a performance benchmarking study of three semiautomatic methods for tumor segmentation, including the two-dimensional (2D) region growing with knowledge-based constraints and the 3D Bayesian rule-based region growing method. Kumar et al. [9] developed a computer-aided diagnosis (CAD) system for tumor segmentation and classification. The alternative fuzzy clustering algorithm was used in the segmentation stage, which used a new distance function instead of the Euclidean metric. Das and Sabut [11] used adaptive thresholding, morphological processing, and a kernelized fuzzy* C*-means (FCM) algorithm to segment liver tumors from CT images. Moghbel et al. [10] proposed an automatic tumor segmentation scheme based on the supervised random walker approach. FCM with cuckoo optimization was applied for the labeling of pixels for the final random walker segmentation.

Active contour approaches, such as fast marching and level set algorithms, are popular segmentation techniques. However, good initialization and speed functions are required in order to obtain accurate segmentation results, especially for tumors with heterogeneous intensities and weak boundaries. Li et al. [12] proposed a new level set model integrating edge- and region-based information with prior information. FCM algorithm was applied for the probabilistic estimation of tumor tissues. Le et al. [13] proposed a semiautomatic method to segment liver tumors from Magnetic Resonance (MR) images, where the fast marching algorithm was used to generate the initial labeled regions, and then other unlabeled voxels were classified by the neural network. Graph cuts methods have also been widely applied for medical image segmentation [14, 15], which can achieve global optimization solution. Stawiaski et al. [14] proposed an interactive segmentation method based on watershed and graph cuts. The method achieved the highest accuracy compared to other semiautomatic or automatic methods in the competition of 2008 Liver Tumor Segmentation Challenge (LTSC08), which was held in conjunction with the Medical Image Computing and Computer Assisted Intervention (MICCAI) 2008 conference [20]. Linguraru et al. [15] presented an automatic tumor segmentation method using graph cuts with Hessian-based shape constraints biased to blob-like tumors. However, the main drawback of such level set or graph cuts based techniques is their relatively high computational cost, especially for 3D volume data.

To deal with the variations of tumor shape, size, intensity, and texture, machine learning based methods were also studied. Foruzan and Chen [19] employed the support vector machine (SVM) and scattered data approximation algorithms to obtain the initial tumor boundary and then refined the final lesion region by using a sigmoid edge model. Kadoury et al. [17] developed an automatic tumor segmentation framework by using the machine learning technique based on discriminant Grassmannian manifolds. Nonlinear intensity distributions of liver and tumor tissues were learned during the training phase. Then, a higher-order conditional random field (CRF) approach was applied to perform the tumor segmentation. Zhang et al. [16] applied watershed transform on the liver region and then employed region-based classification by SVM for tumor segmentation. However, for machine learning based methods, a large amount of training data with gold standards is required during the training process.

Considering the clinical applicability and segmentation accuracy as well as the processing time, our goal was to develop an efficient, robust, and accurate tumor segmentation method with low human interaction. Thus, in this paper, a semiautomatic method based on improved FCM and graph cuts was proposed to realize 3D liver tumor segmentation from CT images. With a single user-selected seed point, both the tumor volume of interest (VOI) and labeled foreground/background regions for graph cuts segmentation were extracted using the confidence connected region growing algorithm. To improve segmentation accuracy, a kernelized FCM algorithm with spatial information was incorporated when estimating the probabilistic models of tumors and liver tissues.

The rest of this paper is organized as follows.  Section 2 introduces the details of the proposed method.  Section 3 presents the experimental results, and the discussion is given in Section 4. Finally, the conclusion is summarized in Section 5.

## 2. Methods

**Figure 1:**
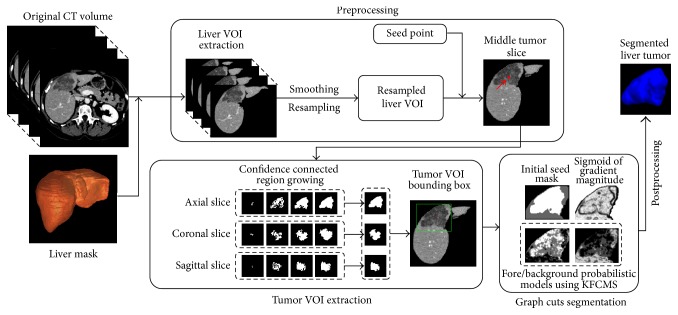
The proposed liver tumor segmentation framework. (VOI: volume of interest; KFCMS: kernelized fuzzy* C*-means with spatial constraints).

The proposed liver tumor segmentation framework is illustrated in Figure 1. It consists of four major steps: (1) preprocessing, (2) tumor VOI extraction, (3) tumor segmentation, and (4) postprocessing. Together with the original input CT volume, the presegmented liver mask is required to extract the liver VOI to reduce the computational cost. The liver mask can be obtained by using a 3D liver segmentation method such as the one in our previous work [21].

### 2.1. Preprocessing

In the preprocessing step, firstly, nonliver tissues were removed and the liver VOI *I*_Liver_ was extracted from the original CT image using the liver mask. Then, *I*_Liver_ was smoothed by median filtering and resampled to isotropic voxel of size 1 × 1 × 1 mm by linear interpolation.

To segment the tumor, one seed point was manually selected on the middle tumor slice. The middle tumor slice was defined as an axial slice, which contained a relatively large lesion region. The seed point *v*_*s*_ was selected near the center of the lesion. To reduce interoperator variability, an automatic correction was applied by replacing the seed point to the darkest voxel within a cube of 5 × 5 × 3 voxels centered at the selected seed point.

### 2.2. Tumor VOI Extraction Using Confidence Connected Region Growing

Based on the seed point, the tumor VOI *I*_Tumor_ was extracted by defining a bounding box around the tumor. Further analysis was conducted on *I*_Tumor_ in order to reduce the computational complexity and processing time. The bounding box was computed using confidence connected region growing algorithm (CCRG), in the axial, coronal, and sagittal slices.

The concept of CCRG is similar to conventional region growing based methods [7, 8]. Through an iterative region growing process that started from the seed point *v*_*s*_, neighboring voxels* v* with intensities inside the defined range *I*_*v*_ ∈ [*m*_*c*_ − *lσ*_*c*_, *m*_*c*_ + *lσ*_*c*_] were included in the tumor region, where *m*_*c*_ and *σ*_*c*_ are the mean and standard deviation of the intensity in the region and* l *is a user-defined multiplicative factor. A region of radius 2 voxels was defined as the initial region. The new inclusion range was acquired by recomputing *m*_*c*_ and *σ*_*c*_ at each iteration of region growing. The expansion continued until no more neighboring voxels were added to the region or the maximum number of iterations was reached.

**Figure 2:**
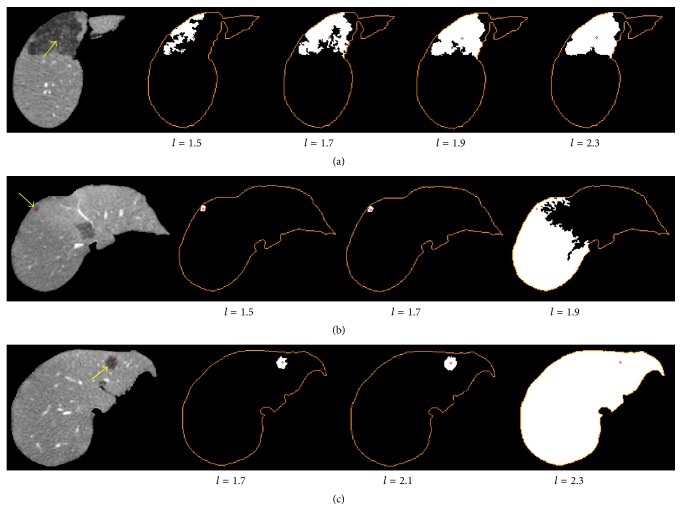
Comparison of confidence connected segmentation results under different values of multiplicative factor* l*. Each row represents an example of segmentation for tumor with varying size. The orange contour indicates liver region and red point shows the tumor seed point.

**Figure 3:**
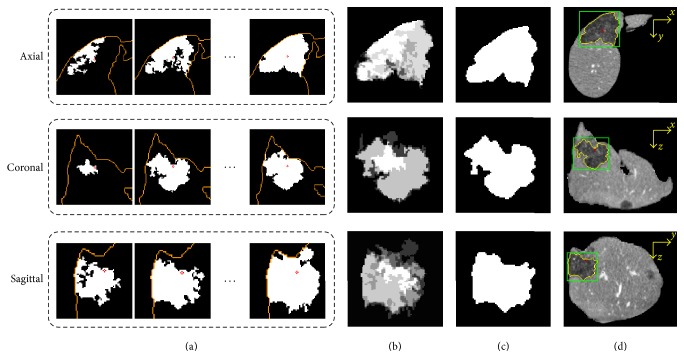
Illustration of tumor VOI extraction using confidence connected region growing. (a) Confidence connected segmentation results under* l* in the range [1.5, 2.5]. (b) The integrated image of (a). (c) The extracted tumor mask. (d) The bounding box of tumor region (green rectangle), the tumor mask (yellow contour), and the seed point (red point).

As shown in Figure 2, the results of confidence connected segmentation were affected by the selection of multiplicative factor* l*. A fixed value of* l* might lead to undersegmentation for large tumors, while causing oversegmentation for small tumors. Thus, to reduce undersegmentation and oversegmentation errors, we employed an iterative process by using confidence connected region growing under the value of* l* ranging from 1.5 to 2.5 (Figure 3). The iteration stopped if the segmented tumor region area was close to that of the liver region, or the distance between the seed point *v*_*s*_ and the centroid of the segmented tumor region was larger than 20 mm. Then, all of the segmented masks were added to generate an integrated image *I*_confi_. The tumor region was then extracted from *I*_confi_ using binary thresholding with the low threshold defined as half of the maximum value in *I*_confi_.

After computing the 3D bounding box of the tumor region on the three cross-section slices, the tumor VOI was defined by adding a safety margin of 15 mm around the 3D bounding box.

### 2.3. Tumor Segmentation Based on Improved Fuzzy* C*-Means and Graph Cuts

#### 2.3.1. Initialization

**Figure 4:**
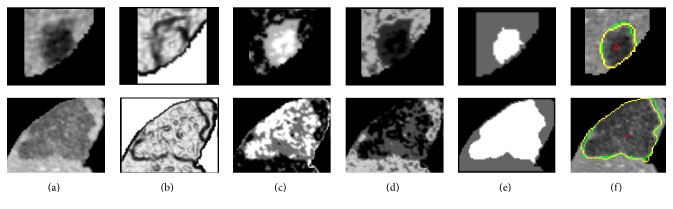
Intermediate results of the initialization of graph cuts segmentation. (a) The middle tumor slice. (b) The sigmoid of gradient magnitude. (c) Probabilistic distribution of lesion. (d) Probabilistic distribution of liver. (e) Initial seed mask. (f) Comparison of segmentation results of the proposed method (yellow contour) and the ground truth (green contour).

The graph cuts segmentation in the tumor VOI *I*_Tumor_ was initialized by the initial seed mask, the sigmoid of gradient magnitude of *I*_Tumor_, and the probabilistic distributions of lesion and liver tissues (Figure 4).

To generate the initial foreground/background seed mask, voxels located inside the confidence connected segmented tumor regions (see Section 2.3) were labeled to 255 as foreground seeds, while the voxels outside the liver region or adjacent to the boundaries of tumor VOI were labeled to 0 as background seeds.

An intensity transform was applied on the gradient magnitude of *I*_Tumor_ according to the following sigmoid function:

(1) $$\begin{array}{c} I_{sg}={\left(\;1+\exp\left(\;-\frac{\left(I_{g}-\beta_{s}\right)}{\alpha_{s}}\;\right)\;\right)}^{-1}, \end{array}$$

where *I*_*g*_ denotes the gradient magnitude of *I*_Tumor_, *I*_sg_ is the sigmoid of *I*_*g*_, *α*_*s*_ and *β*_*s*_ are parameters that define the mapping range of the input *I*_*g*_, *α*_*s*_ determines the input intensity window width, and *β*_*s*_ defines the window center.

Gaussian mixture model (GMM) is commonly applied to estimate the intensity distribution [21, 22]. However, only intensity information is used in GMM without considering spatial information. In this paper, a kernelized FCM algorithm with spatial information was employed to estimate the probabilistic models of lesion/liver tissues.

#### 2.3.2. Kernelized FCM with Spatial Constraints

Let *I* = (*I*_1_, *I*_2_,…, *I*_*N*_*v*__) denote the intensities of the voxels in the tumor VOI and let *C* = (*c*_1_, *c*_2_,…, *c*_*N*_*c*__) denote the cluster centers, where *N*_*v*_ and *N*_*c*_ are the number of voxels and cluster centers, respectively. Fuzzy clustering based algorithms assign voxels* v* to each cluster by minimizing the objective function defined as follows [23, 24]:

(2) $$\begin{array}{c} J=\overset{N_{v}}{\underset{j=1}{\sum }}\;\overset{N_{c}}{\underset{i=1}{\sum }}u_{ij}^{m_{f}}{\left\|\;I_{j}-c_{i}\;\right\|}^{2}, \end{array}$$

where *u*_*ij*_ is a partition matrix that represents the membership of the voxel in the* i*th cluster and *m*_*f*_ is a weighting factor that controls the fuzziness of the resulting partition. The objective function is minimized through an iterative optimization. The membership value in *u*_*ij*_ indicates the probability that a voxel belongs to a specific cluster.

In traditional FCM algorithm, the membership matrix and cluster centers were updated by

(3) uij=∑k=1NcIj−ciIj−ck2/mf−1−1,ci=∑j=1NvuijmfIj∑j=1Nvuijmf.

Only intensity information was included without considering the local spatial information. To improve the robustness of fuzzy classification, spatial neighborhood information was incorporated into the membership matrix [24], and the Euclidean distance was replaced by the Gaussian kernel-induced distance measure [23, 25]. The membership matrix was then updated by

(4) $$\begin{array}{c} u_{ij}^{\prime }=\frac{u_{ij}^{p}h_{ij}^{q}}{\sum_{k=1}^{N_{c}}u_{kj}^{p}h_{kj}^{q}}, \end{array}$$

where

(5) hij=∑k∈NBIjuik,uij=1−KIj,ci−1/mf−1∑k=1Nc1−KIj,ck−1/mf−1,KI,c=exp⁡−I−c22σf2.

Here, *p* and *q* are weighting factors determining the influence of both functions, NB(·) represents the neighboring window, and *σ*_*f*_ is the adjustable parameter of the Gaussian kernel *K*(*I*, *c*). The new cluster centers were updated by

(6) $$\begin{array}{c} c_{i}=\frac{\sum_{j=1}^{N_{v}}u_{ij}^{\prime m_{f}}K\left(I_{j},c_{i}\right)I_{j}}{\sum_{j=1}^{N_{v}}u_{ij}^{\prime m_{f}}K\left(I_{j},c_{i}\right)}. \end{array}$$

For convenience, the improved kernelized FCM algorithm with spatial constraints was called KFCMS. Details of the KFCMS algorithm are presented in Algorithm 1.

As shown in Figures 4(c) and 4(d), the probabilistic models of lesion and liver were obtained using KFCMS. The probability value of each voxel in tumor VOI was in range [0,1] and was mapped to grayscale for visualization.

#### 2.3.3. Graph Cuts Segmentation

Tumor segmentation problem can be formulated in terms of energy minimization, and graph cuts segmentation is an optimization process aimed to find optimal surface with minimal cost [26, 27]. In this paper, a graph was constructed on the tumor VOI, which consisted of three types of nodes *𝒱* = {*𝒯*, *𝒩*, *𝒮*} and three types of undirected edges *ℰ* = {*E*_*n*_, *E*_*t*_, *E*_*s*_}. The sink node *𝒯* represents a set of foreground seeds, and the source node *𝒮* denotes the background seeds. The node set *𝒩* corresponds to the voxels in *I*_Tumor_. In the edge set *E*_*n*_, the so-called n-link edges connect all neighboring voxels. In *E*_*t*_ and *E*_*s*_, the so-called* t*-link edges connect each voxel to the sink and source node, respectively.

For the node set *𝒩* and a set of labels *ℒ*, the goal is to find a labeling *f* : *𝒩* → *ℒ* by minimizing the following energy function *E*(*f*):

(7) $$\begin{array}{c} E\left(\;f\;\right)=\underset{v\in N}{\sum }R\left(\;f_{v}\;\right)+\gamma \underset{\left(v,u\right)\in E_{n}}{\sum }B\left(\;f_{v},f_{u}\;\right), \end{array}$$

where *R*(*f*_*v*_) and *B*(*f*_*v*_, *f*_*u*_) are the region term and boundary term, respectively, and *γ* is a weighting factor. These terms correspond to the weights of the two types of edges *ℰ* and are defined as follows:

For  (*v*, *𝒯*) ∈ *E*_*t*_,
  (8) $$\begin{array}{c} R\left(\;f_{v}\;\right)=\left\{\begin{array}{cc} \infty , & v\in T,\\ 0, & v\in S,\\ P_{fg}\left(v\right), & \text{others.} \end{array}\right. \end{array}$$
For  (*𝒮*, *v*) ∈ *E*_*s*_,
  (9) $$\begin{array}{c} R\left(\;f_{v}\;\right)=\left\{\begin{array}{cc} 0, & v\in T,\\ \infty , & v\in S,\\ P_{bkg}\left(v\right), & \text{others.} \end{array}\right. \end{array}$$

The regional term *R*(*f*_*v*_) specifies the cost of assigning a label to voxel* v* based on the probabilistic models *P*_fg_(*v*) and *P*_bkg_(*v*) of tumor and liver tissues (see Section 2.3.2):

(10) Bfv,fu=δfv,fu·Iv−Iu2+1−1+λIsg,δfv,fu=1,if fv≠fu,0,if fv=fu.

The boundary term *B*(*f*_*v*_, *f*_*u*_) represents the penalty of discontinuity between two adjacent voxels* v* and* u* by integrating both the intensity and gradient information *I*_sg_ (see Section 2.3.1).

After the graph was constructed, the optimal tumor surface can be obtained via the max-flow/min-cut algorithm [26, 28]. Details of the graph cuts algorithm are presented in Algorithm 2.

### 2.4. Postprocessing

After completing the graph cuts segmentation, postprocessing steps were conducted to refine the segmentation result, including connected region selection by the seed point *v*_*s*_, median filtering, and morphological dilating. Then, the postprocessed result was resampled to the original input CT resolution and size for further evaluation.

## 3. Experimental Results

### 3.1. Datasets

The proposed method was evaluated on the public clinical contrast-enhanced CT dataset (3Dircadb (http://www.ircad.fr/research/3dircadb)), which contains 15 CT volume images involving 120 liver tumors of different sizes. The pixel spacing, slice thickness, and number of slices varied from 0.56 to 0.87 mm, 1 to 4 mm, and 74 to 260, respectively, with the in-plane resolution of 512 × 512 pixels in all cases. Tumors manually segmented by clinical experts were also provided and considered as ground truth. Tumors less than 5 mm in diameter were not included in our experiment, as they are too small and only visible in one or two slices, which are not suitable for 3D segmentation. As our method was designed for the segmentation of single tumors, connected tumors were also excluded.

### 3.2. Evaluation

To evaluate the performance of the proposed method quantitatively, the five metrics used in the competition of LTSC08 [20] and Dice similarity coefficient (DICE) were computed to measure the volumetric overlap or surface distance of the segmentation result compared to ground truth. The five metrics are volumetric overlap error (VOE), relative volume difference (RVD), average symmetric surface distance (ASD), root mean square symmetric surface distance (RMSD), and maximum symmetric surface distance (MaxD). For the detailed definitions of these statistic measures, please refer to [29, 30]. The value of DICE ranges from 0 to 1, with a value of 0 indicating no overlap and 1 representing perfect segmentation. For the other five metrics, a value of 0 represents perfect segmentation. A negative value of RVD indicates undersegmentation. A scoring system, employed in LTSC08, assigned a score between 0 and 100 to each metrics to compute an overall score. A score of 100 indicates a perfect tumor segmentation, and the manual segmentation of the average quality (VOE = 12.94%, RVD = 9.64%, ASD = 0.40 mm, RMSD = 0.72 mm, and MaxD = 4.0) is worth the score of 90.

In our experiments, the parameters described in Section 2.3 were determined experimentally by considering the segmentation performance and computational cost. The proposed method was implemented in C++ and tested on a 2 GHz Intel Xeon E5-2620 PC workstation with 32 GB RAM.

### 3.3. Results

**Figure 5:**
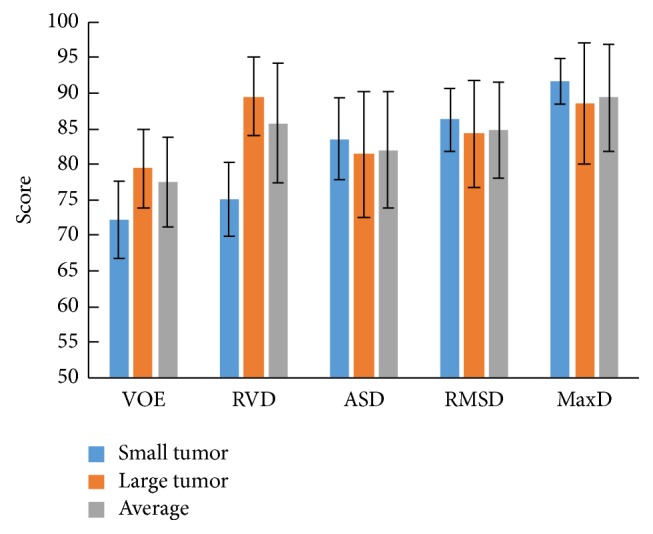
Scores of segmentation results obtained by the proposed method (VOE: volumetric overlap error; RVD: relative volume difference; ASD: average symmetric surface distance; RMSD: root mean square symmetric surface distance; MaxD: maximum symmetric surface distance).

The statistical results of the proposed method are represented in Table 1. In this paper, a tumor with an approximate diameter larger than or equal to 10 mm was defined as a large one, and a small tumor had a diameter less than 10 mm. Our method achieved an average VOE and RVD of 29.04% and −2.20%, an average ASD, RMSD, MaxD of 0.72 mm, 1.10 mm, and 4.25 mm, and an average DICE of 0.83, respectively.  Figure 5 shows the scores of our method for small tumor and large tumor segmentation. The average score of VOE for all tumors was 77.56. For large tumors, a VOE of 26.62%, RVD of −3.11%, ASD of 0.74 mm, MaxD of 4.57 mm, and DICE of 0.84 were obtained. For small tumors, a higher VOE of 35.90% and a lower DICE of 0.78 were obtained, which led to lower accuracy scores.

As shown in Table 2, the average running time of the proposed method taken to do seed point selection, tumor VOI extraction, and graph cuts segmentation was 45 s. For a particular tumor, the running time varied with the tumor size. For a large tumor with approximately 45 mm in diameter, the time for VOI extraction and graph cuts segmentation was 3 s and 28 s, respectively. The time for selecting the middle tumor slice and the tumor seed point was typically less than 30 s.

Table 2 shows the comparative results of the proposed method with previous methods [10, 12, 14, 17, 19]. The methods of Li et al. [12], Stawiaski et al. [14], and Kadoury et al. [17] were evaluated on the dataset provided by the competition of LTSC08, which included 4 CT volumes with 10 tumors. However, the LTSC08 dataset is not available now. The methods of Moghbel et al. [10] and Foruzan and Chen [19] were evaluated using the 3Dircadb dataset involving 120 tumors. The 3Dircadb dataset includes more challenging cases than the LTSC08 dataset does. Compared with other previous methods, our method improved the time efficiency of liver tumor segmentation, while maintaining accuracy.

**Figure 6:**
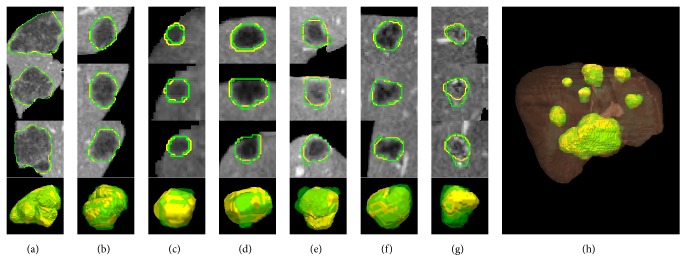
Segmentation results obtained by the proposed method compared to ground truth manual segmentation. Each column from (a) to (g) shows one case of tumor segmentation results in axial, coronal, and sagittal view and the 3D reconstruction result. (h) 3D reconstruction of segmented tumors inside the liver. The contour/surface of the ground truth is in green. The contour/surface of the segmented tumor by the proposed method is in yellow. The liver surface is shown in brown.

**Figure 7:**
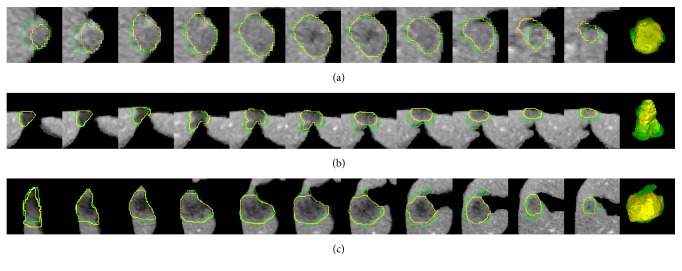
Some challenging cases. Each row shows a case of tumor segmentation results in sequential slices and 3D visualization. The contour/surface of the ground truth is in green. The contour/surface of the segmented tumor by the proposed method is in yellow.

Figure 6 shows a typical example of one patient's CT volume data consisting of 7 tumors with different sizes, intensities, and positions. It can be seen that our method achieved comparable results to the ground truth manual segmentation in most cases. However, relatively large undersegmentation and oversegmentation errors occurred when the tumor presented ambiguous or low contrast boundaries (Figures 6(e) and 6(g)). More examples of challenging cases are presented in Figure 7.

## 4. Discussion

In this paper, we proposed a new semiautomatic method to segment liver tumors from CT volume images. It consists of two major steps: (1) tumor VOI extraction using confidence connected region growing algorithm with one single seed point; (2) tumor segmentation based on KFCMS and graph cuts algorithms.

The proposed method was evaluated on the 3Dircadb dataset. Experimental results showed that the segmentation accuracy of our method was comparable to that of the manual segmentation for most cases (Figure 6), while the efficiency of segmentation was improved significantly with an average processing time less than one minute per tumor (Table 2). As shown in Table 1 and Figure 5, small tumors were prone to have higher volumetric overlap errors than large tumors. This was because, for small tumors, a small discrepancy between the segmentation and the ground truth could lead to a considerably increased error. Figure 7 shows the undersegmentation and oversegmentation results on challenging cases, which had lower accuracy scores than average. The undersegmentation and oversegmentation errors were mainly due to the low contrast or weak boundaries of tumors. Large tumors with inhomogeneous intensity adjacent to the boundary of liver were also prone to be undersegmented.

Compared with previous approaches (Table 2), the proposed method can achieve accurate 3D liver tumor segmentation in a fast manner. Among the graph based methods, Stawiaski et al. [14] constructed a region adjacency graph on subregions of watershed transform and applied graph cuts for tumor segmentation; Kadoury et al. [17] applied a high-order graphical model with potential functions based on discriminant Grassmannian manifolds learning. Kadoury's method achieved one of the highest accuracies in Table 2, and the average segmentation time per tumor was 102 s, with the average training time of 6 hours. For Staviaski's method, it took a long time to segment a tumor, including 1 minute to define the tumor VOI manually, 1-2 minutes to mark seed points for graph cuts segmentation in three orthogonal slices, and 2–5 minutes to refine the segmented tumor contours in an interactive way. Unlike Staviaski's and Kadoury's methods, user interaction was reduced to mark one single seed point in our method, and then the tumor VOI extraction and graph cuts initialization processes were conducted automatically and efficiently. Fuzzy probabilistic models and gradient information were introduced to improve the accuracy and robustness of our method.

Among the semiautomatic methods, Li et al. [12] used level sets method integrating FCM based regional information and gradient based boundary information; Foruzan and Chen [19] obtained initial tumor contours using supervised watershed, SVM, and scattered data approximation for large/small tumors and then refined the contours based on sigmoid edge model. For Li's method, an average time of 30 s was taken per tumor in a given tumor VOI, which was defined by two user-selected seed points in the tumor and surrounding liver regions. The average time of Foruzan's method was 154 s per tumor, including seed points selection, training, tumor contour extraction, and refinement. Several seed points for the tumor, liver, and other background were marked in the middle tumor slice through user interaction [19]. Compared with Li's and Foruzan's methods, we employed confidence connected region growing with one single seed point in three orthogonal slices to extract the tumor VOI rapidly. The kernelized FCM with spatial constraints, instead of FCM, and the sigmoid of gradient magnitude were incorporated in the energy function as regional and edge information. No training process was needed for the initialization of our graph cuts based method.

Among all the methods in Table 2, Moghbel et al. [10] proposed an automatic hybrid method based on FCM with cuckoo optimization and random walkers. It achieved the highest accuracy and the average time was 30 s per slice, approximately 16 minutes per case. The performance of our method on the 3Dircadb dataset was comparable to that of Foruzan, and the tumor segmentation procedure was performed in a fast way with low computational cost.

The contributions of the proposed method are as follows. (1) User interaction was reduced significantly compared to the traditional graph cuts method. Only one seed point was required to be marked close to the center of tumor in the middle tumor slice. An automatic correction was also conducted to reduce interoperator variability in choosing the seed point. (2) The tumor VOI was extracted adaptively using confidence connected region growing in the three orthogonal slices to reduce computational cost. Initial tumor regions on the three slices were obtained based on the integrated images of CCRG segmentations to improve the robustness of tumor VOI extraction. (3) Image intensity and spatial and gradient information, corresponding to the probabilistic distributions estimated by KFCMS and the sigmoid of gradient magnitude, were incorporated in the energy function to improve the performance of graph cuts segmentation. (4) The proposed method was efficient while maintaining accuracy. Such a fast segmentation method might be used for practical applications like trajectory planning for radiofrequency ablation without significantly increasing the processing time, which involves segmentations of all relevant organs/structures [31].

One limitation of our method is that it mainly focused on liver tumors with hypointensity. For tumors with hyperintensity, some parts of the method shall be adjusted like the automatic correction of seed point in the VOI extraction step. Also, the evaluation experiments should be extended to include more types of liver lesions. To deal with challenging cases of tumors with low contrast and ambiguous boundaries, our method may be enhanced by incorporating more texture information and a contour refinement process in future.

## 5. Conclusion

This paper presented a semiautomatic method for 3D tumor segmentation in CT images using improved FCM and graph cuts algorithms. Low interaction was required and the tumor VOI was extracted to reduce computational cost. Probabilistic distributions estimated by KFCMS and sigmoid of gradient magnitude were incorporated into the energy function of graph cuts. While being faster than most of previous methods with an average computational time of 45 s per tumor, the proposed method achieved accurate segmentation results. In future, improvements for reducing undersegmentation and oversegmentation errors shall be conducted to enhance the performance of the proposed method on challenging cases. Datasets with more types of tumors for evaluation are needed.

## Figures and Tables

**Algorithm 1:**
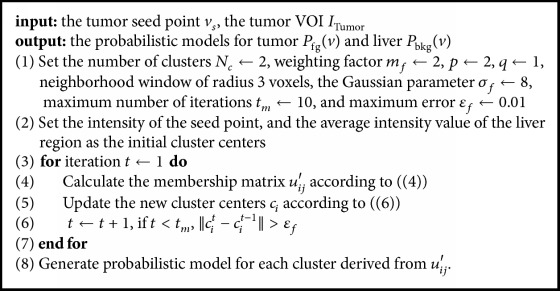
Kernelized FCM with spatial constraints (KFCMS).

**Algorithm 2:**
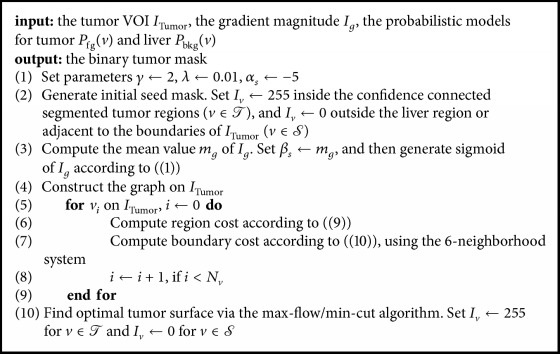
Graph cuts segmentation.

**Table 1:** Quantitative evaluation of the proposed tumor segmentation method. Results are represented as mean ± standard deviation (VOE: volumetric overlap error; RVD: relative volume difference; ASD: average symmetric surface distance; RMSD: root mean square symmetric surface distance; MaxD: maximum symmetric surface distance; DICE: Dice similarity coefficient).

	VOE (%)	RVD (%)	ASD (mm)	RMSD (mm)	MaxD (mm)	DICE	Diameter (mm)
*Small tumor*	35.90 ± 7.06	0.38 ± 25.29	0.66 ± 0.23	0.99 ± 0.31	3.34 ± 1.27	0.78 ± 0.05	9.42 ± 2.11
Max	47.76	37.99	1.25	1.74	6.32	0.86	14.80
Min	24.11	−31.24	0.40	0.69	2.00	0.69	5.54
*Large tumor*	26.62 ± 7.11	−3.11 ± 11.05	0.74 ± 0.35	1.13 ± 0.54	4.57 ± 3.40	0.84 ± 0.05	16.95 ± 9.20
Max	46.50	18.25	2.40	3.27	20.78	0.95	45.38
Min	8.78	−18.07	0.40	0.68	1.73	0.70	7.61
*Average*	29.04 ± 8.16	−2.20 ± 15.88	0.72 ± 0.33	1.10 ± 0.49	4.25 ± 3.03	0.83 ± 0.06	14.99 ± 8.63

**Table 2:** Statistical performance of the proposed method compared to some other methods. Results are represented as mean ± standard deviation.

Method	Year	Auto	Dataset	VOE (%)	RVD (%)	ASD (mm)	RMSD (mm)	MaxD (mm)	DICE	Runtime
Stawiaski et al. [14]	2008	Interactive	LTSC08	29.49 ± 12.80	23.87 ± 34.72	1.50 ± 0.67	2.07 ± 0.89	8.30 ± 4.10	—	5–8 mins
Li et al. [12]	2012	Semi	LTSC08	26.31 ± 5.79	−10.64 ± 7.55	1.06 ± 0.38	—	8.66 ± 3.17	—	30 s
Kadoury et al. [17]	2015	Auto	LTSC08	25.2 ± 1.7	14.3 ± 2.8	1.4 ± 0.3	1.6 ± 0.4	6.9 ± 1.8	—	102 s
Moghbel et al. [10]	2016	Auto	3Dircadb	22.78 ± 12.15	8.59 ± 18.78	—	—	—	0.75 ± 0.15	30 s/slice
Foruzan and Chen [19]	2016	Semi	3Dircadb	30.61 ± 10.44	15.97 ± 12.04	4.18 ± 9.60	5.09 ± 10.71	12.55 ± 17.07	0.82 ± 0.07	154 s
*Our method*		Semi	3Dircadb	29.04 ± 8.16	−2.20 ± 15.88	0.72 ± 0.33	1.10 ± 0.49	4.25 ± 3.03	0.83 ± 0.06	45 s
